# Providing Emotional Support During the Process of Multiple Sclerosis Diagnosis (PrEliMS): A Feasibility Randomised Controlled Trial

**DOI:** 10.1177/02692155241284781

**Published:** 2024-09-25

**Authors:** Roshan das Nair, Jacqueline R Mhizha-Murira, Gogem Topcu, Tierney Tindall, Clare Bale, Nima Moghaddam, Grit Scheffler-Ansari, Avril Drummond, Deborah Fitzsimmons, Nikos Evangelou

**Affiliations:** 1 School of Medicine, 6123 University of Nottingham, Nottingham, UK; 2 Institute of Mental Health, Nottinghamshire Healthcare NHS Foundation Trust, Nottingham, UK; 3 Health Division, SINTEF, Trondheim, Norway; 4 Multiple Sclerosis Patient and Public Involvement Group, Nottingham, UK; 5 School of Psychology, University of Lincoln, Lincoln, UK; 6 School of Health Sciences, 6123 University of Nottingham, Nottingham, UK; 7 Swansea Centre for Health Economics, Swansea University, Swansea, UK

**Keywords:** Multiple sclerosis, diagnosis, emotional support, psychological adjustment, peer support

## Abstract

**Objectives:**

To evaluate the feasibility and acceptability of an emotional support programme for newly diagnosed people with multiple sclerosis.

**Design:**

Three-arm, mixed methods, randomised controlled trial comparing usual care, versus usual care plus nurse-specialist support, versus usual care plus nurse-specialist support plus peer support.

**Participants:**

Community-dwelling adults within two years of diagnosis *or* undergoing diagnosis.

**Interventions:**

PrEliMS involves information provision, emotional support, and strategies and techniques based on psychoeducation, Acceptance and Commitment Therapy principles, supportive listening. One version of the intervention was provided by nurse-specialists alone and the other was provided by nurse-specialists plus peer support.

**Main measures:**

The main outcome of interest was the feasibility of proceeding to a definitive trial, exploring recruitment rate, acceptability, completion of outcome measures (perceived stress, mood, self-efficacy, psychological impact, and service use), and signal of efficacy.

**Results:**

Of 40 participants randomised (mean age 36.2 years (SD = 14.8); 54% women; 85% with relapsing-remitting MS), 36 and 38 returned 3- and 6-month questionnaires, respectively. Participant interviews suggested the trial was largely feasible, and the intervention acceptable, with some amendments to trial procedures and intervention delivery noted. There were, however, no statistically significant differences between groups at followup for any measures, and effect-size estimates were small.

**Conclusion:**

A definitive trial combining nurse-specialist and peer support adjustment to diagnosis intervention is warranted, but more work exploring the delivery and fidelity of the intervention is needed before this is pursued

## Introduction

Being diagnosed with multiple sclerosis can be stressful and psychologically demanding for patients and their families^1^; with people with multiple sclerosis describing the process as confusing and frustrating, eliciting feelings of anxiety, grief, anger, fear, and distress.^1,2^ These issues may be due to the unpredictable nature of multiple sclerosis, lack of a single diagnostic biomarker, and inconsistent service delivery. Therefore, the importance of providing accessible information, advice, and support at diagnosis is well recognised in both UK National Institute for Health and Care Excellence (NICE) guidelines^3^ and the European Multiple Sclerosis Platform code of practice.^4^

Challenges faced during the diagnostic phase influence patients’ perceptions of multiple sclerosis and their future relationship with healthcare teams.^1,5^ This is particularly important for rehabilitation, because many of the symptoms of multiple sclerosis require long-term input from rehabilitation specialists. Consequently, how this phase is managed may influence patients’ adjustment to multiple sclerosis later; so supporting people with multiple sclerosis adequately around the diagnosis process is crucial. However, poor emotional support and information provision around this period is common.^2,6^

Given this, and the lack of stakeholder *co-constructed* emotional support programmes being delivered in the UK for people with multiple sclerosis around diagnosis, we developed the “Providing emotional support around the multiple sclerosis diagnosis process” (PrEliMS) interventions. These were developed based on evidence from our two systematic reviews,^2,6^ focus groups with relevant stakeholders (people with multiple sclerosis, family members/carers and multiple sclerosis clinicians),^7^ Patient and Public Involvement input, clinical experiences, and the research teams’ expertise in designing complex emotional support interventions.

In line with guidance for developing and evaluating complex interventions,^8^ before conducting a definitive trial we aimed to evaluate key feasibility parameters: (1) feasibility and acceptability of trial procedures, intervention, and newly developed service pathway (2) intervention fidelity, and (3) outcome parameters to undertake a clinical- and cost-effectiveness analysis for a future randomised controlled trial (RCT).

## Methods

Ethical approval was granted by the Health Research Authority London (Bloomsbury) Research Ethics Committee (18/LO/1468) and was prospectively registered (ClinicalTrials.gov NCT03735056). The study Sponsor was the University of Nottingham.

Participants were recruited from multiple sclerosis clinics at a UK National Health Service (NHS) Trust hospital outpatient neurology department between November 2018 and April 2020. A neurologist or multiple sclerosis nurse-specialist (henceforth referred to as ‘nurse specialist’) introduced the study to eligible patients during their clinic appointments and consent was obtained for a researcher to contact them. Patients were provided with an information pack by post or email and further screened for eligibility by the researchers. Eligible participants were: within two years of their multiple sclerosis diagnosis *or* were undergoing diagnosis process; aged ≥18 years, able to communicate in English and provide consent. We included both those who were recently diagnosed and those who were undergoing diagnosis process because multiple sclerosis diagnosis is a complex process that occurs over several months to several years. Patients were excluded if they had a severe co-morbid psychiatric condition, and were receiving or had received psychological interventions within the previous three months. Eligible participants completed consent and baseline assessments online, over the telephone with a researcher or by post according to their preference (see Table 1).

**Table 1.** Measures and data collection timepoints.

Measure	Domain assessed	Data collection timepoint		
		Baseline	3-month follow-up	6-month follow-up
Demographics questionnaire (including information on their MS diagnosis)	Age, gender, ethnicity, highest education level attained, employment status, duration of MS diagnosis, how long it took to receive multiple sclerosis diagnosis	x		
Perceived Stress Scale 4-item (PSS-4)^9^	Level of perceived stress	x	x	x
Hospital Anxiety and Depression Scale (HADS)^10^	Level of mood disturbance	x	x	x
Multiple Sclerosis Impact Scale-29 (MSIS-29)–psychological subscale (MSIS-psych) (Rasch, version 2)^11^	Perceived psychological impact of MS	x	x	x
Multiple Sclerosis Self-Efficacy Scale (MSSE)^12^	Extent to which participants feel in control of their condition.	x	x	x
The EuroQol 5 Dimension 5 Level (EQ-5D-5L)^13^	Generic patient-reported measure of health-related quality of life	x	x	x
Bespoke service use questionnaire (adapted from a previous multi-centre multiple sclerosis trial)^14^	Use of health and social services	x	x	x

MS: multiple sclerosis.

Participants were then randomly allocated to usual care (Control), or usual care *plus* nurse-specialist support (Intervention 1), or usual care *plus* nurse-specialist *and* peer support (Intervention 2) on a 1:1:1 ratio. A pre-defined pseudo-random list, with block sizes of 3, 6 and 9, was generated by an independent, centralised online randomisation service (www.sealedenvelope.com), and maintained by the trial manager. Given the nature of the intervention, participants and intervention providers (nurse-specialist and peer support workers) could not be blinded. The researchers collecting outcome data (who were psychologists with Masters or post-doctoral training) and the researchers conducting the statistical analyses were blinded to treatment allocation.

We aimed to randomise up to 60 participants (20 participants per group), to offer sufficient information to inform the design of a Phase III RCT, as 10–20 per group is the recommended sample size for feasibility trials for standardised small (0.2) or medium (0.5) effect sizes.^15^

### Interventions

PrEliMS is multi-faceted, involving various components and a range of strategies and techniques. It is person-based, underpinned by the conceptual understanding of adjustment to multiple sclerosis diagnosis.^2^ This posits that providing resources and coping strategies during the diagnosis process enhances adjustment to diagnosis (e.g., by reducing negative emotional responses, improving management techniques).^6^ A description of the intervention is presented using the recommended Template for Intervention Description and Replication checklist^16^ in Supplementary Material 1. There are two PrEliMS interventions:

Intervention 1: Nurse-specialists provided standardised emotional support and advice to patients at diagnosis to establish and help sustain coping strategies. Participants received a one-to-one, face-to-face session in clinic, via videoconferencing, or telephone within two weeks of diagnosis. These calls were to be arranged as close to the 2-week post-diagnosis period as determined by the stakeholder-informed new service pathway; and for those who were diagnosed earlier, as soon as they were referred to the study. Sessions were to last up to 90 min and included answering questions about multiple sclerosis, providing psychoeducation, teaching Acceptance and Commitment Therapy-based strategies,^17^ and referring to other services (where needed). Participants were provided with an Acceptance and Commitment Therapy-based self-help book (“Better living with a diagnosis of multiple sclerosis: Patient Workbook”). Additional support sessions, if required, were provided over phone. Nurse-specialists were trained and supervised by clinical psychologists (RdN and NM). Group-based training was delivered in a half-day session, with a 60-min refresher session offered mid-trial. They received hour-long monthly supervision sessions from NM.

Intervention 2: Comprised Intervention 1 *plus* peer support. Peer support uses supportive listening to provide the opportunity to talk freely about experiences, including thoughts and feelings about diagnosis, in a non-judgmental, safe environment. Participants received a minimum of two sessions with a peer support worker (someone with multiple sclerosis or a family member or carer of a person with multiple sclerosis), recruited from local multiple sclerosis charity branches. Peer support workers were trained and supported throughout the trial (as needed) by RdN and a post-doctoral researcher in health psychology (GT). Peer support sessions lasting up to 60 min were face-to-face or via telephone/videoconferencing, after the nurse-specialist support session, 2–6 weeks following diagnosis.

Participants in the control group received their usual clinical care from the multiple sclerosis clinics as per NICE guidelines, which recommends first appointment with multiple sclerosis Nurse Specialist to occur within 6 weeks of diagnosis.^3^ Typically, this includes more information about what multiple sclerosis is, and the disease modifying therapies available and the pros and cons of each.

Participants in all groups were assessed 3- and 6-months post-randomisation using the measures outlined in Table 1, either online or by post.

The intervention fidelity (Intervention 1 and 2) was assessed through: (1) Session record forms completed by nurse-specialists and peer support workers (detailing topics discussed and information provided); (2) Time-sampling of audio-recordings of nurse-specialist support sessions.

Two researchers (JMM and GSA) conducted brief semi-structured interviews between the two follow-up periods with intervention providers (nurse-specialists and peer support workers) and people with multiple sclerosis (up to seven from each group). Both researchers were involved in other aspects of the trial (e.g., recruitment and data collection). Patient participants were sampled using a purposive, maximum-variation sampling strategy^18^ to ensure a variety of participants in terms of demographics (e.g., age, gender) and clinical characteristics (e.g., multiple sclerosis type) to assess acceptability of intervention and trial procedures. The interview schedules were developed with patient and public involvement partners (See Supplementary Material 2). Interviews were audio-recorded and transcribed verbatim.

The health economic evaluation focused on establishing the main cost drivers, necessary parameters, and suitable framework to undertake a full cost-effectiveness analysis in a future trial.

The Trial Management Group categorised the findings based on guidance for progression criteria to definitive trials,^19^ to arrive at Red-Amber-Green ratings for each key feasibility outcome. The process for decision-making followed the ADePT framework^20^ for identifying solutions to the issues identified.

A detailed description of the outcomes, how these mapped onto the aims of the feasibility study and how they were assessed can be found in Supplementary Material 3.

For quantitative data, analyses were conducted on an intention-to-treat basis using SPSS v25. Descriptive statistics were used to characterise the sample and to indicate retention and progression of participants through the trial. For effect-size estimation and sample size calculations for a definitive trial, multiple one-way analyses of variance (ANOVAs) were conducted to compare the different groups on all outcome measures at each follow-up. The Reliable Change Index method^21^ was used to assess whether individual changes between baseline and follow-up were greater than that expected by chance and clinically significant.

Time sampling enabled us to determine whether the interventions were delivered according to the manual. Each one-minute unit of the audio data was coded using a coding scheme identifying the key intervention components, content of discussions was documented as either related to the intervention (patient-cued, based on the needs assessment) or unrelated. The initial coding frame was developed by the research team based on a consensus regarding what was judged to be the key components of the interventions. Additional codes were developed iteratively in an inductive manner by JMM and through discussion with the research team. The primary activity of the individual speaking (nurse-specialist or patient-participant) was also documented. To assess intervention fidelity, audio-recordings of nurse-specialist support sessions were rated to determine to what extent intervention delivery was congruent with the underpinning approach to emotional support (e.g., openness to difficult experiences and engagement in valued actions). The final coding framework had 12 items, 11 of which were scored as being congruent to the Acceptance and Commitment Therapy model (i.e., consistency with Acceptance and Commitment Therapy principles in the workbook; we used definitions from the validated Acceptance and Commitment Therapy Fidelity Measure).^22^ These were scored 0 to 3 (No; Yes – somewhat; Yes – mostly; Yes – fully). The one item that documented incongruence was reversed and scored 0 to 2 (Yes – fully; Yes – somewhat; No). Therefore, the total possible score was 35. There was no threshold for determining fidelity, and these scores were used descriptively.

For qualitative data, anonymised transcripts were analysed on NVivo v12 following framework analysis.^23^ For each participant group, the interview guide (based on the trial aims) informed the development of the initial thematic framework.

## Results

**Figure 1.**
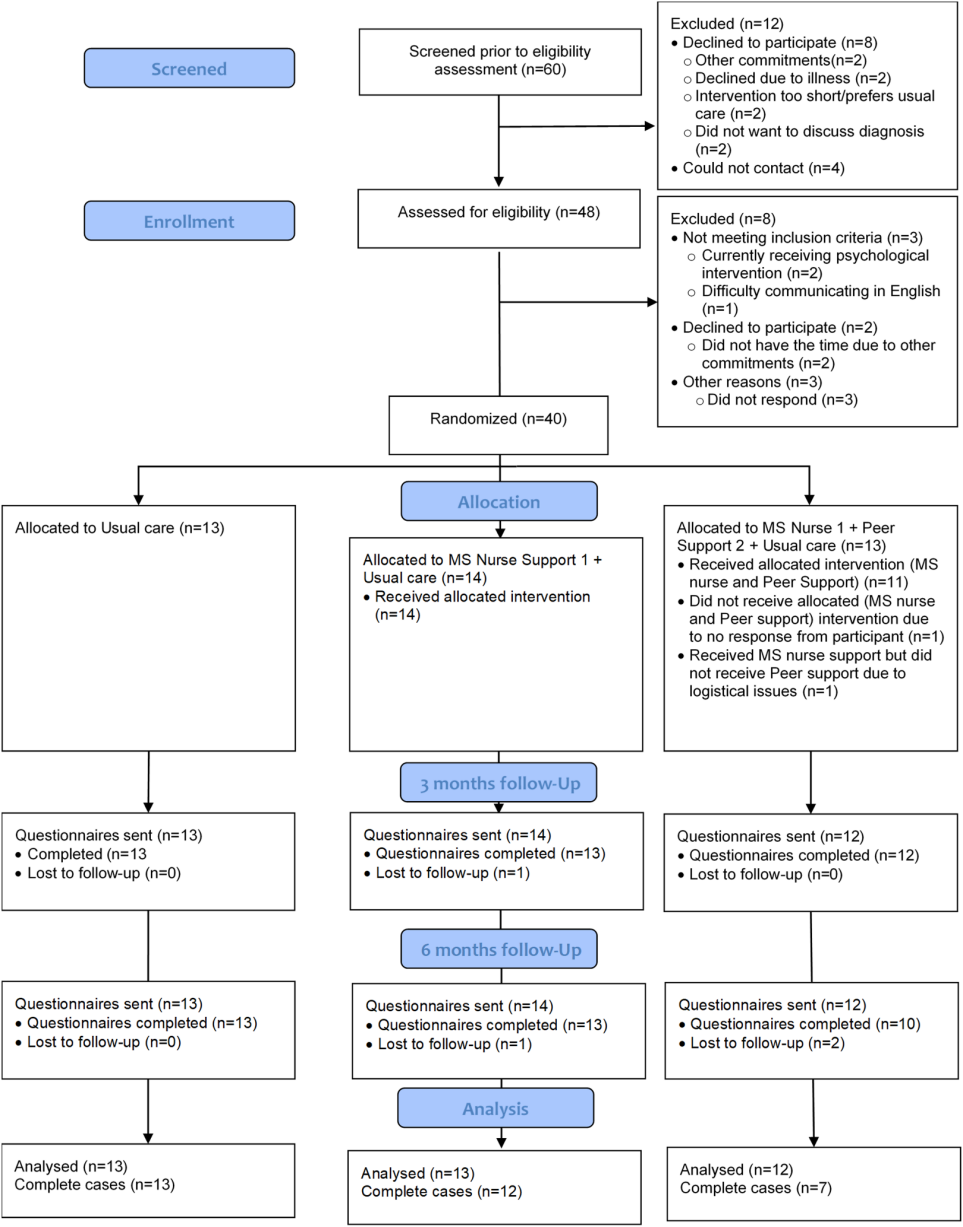
CONSORT flow diagram.

Forty people were recruited and randomised (see CONSORT, Figure 1) over 18 months. The groups were well-matched on demographic and clinical characteristics (Table 2). There were fewer men and people with relapsing-remitting multiple sclerosis in Intervention 2, and more people were in employment in Intervention 1, but these differences were not statistically significant. There were no statistically significant baseline differences between the groups for health-related quality of life (EQ-5D-5L).

**Table 2.** Participant demographic and clinical characteristics.

		Usual care (n = 13)			Nurse-specialist support 1 (n = 14)			Nurse-specialist and Peer support 2 (n = 13)		
		*n*	Mean	SD	*n*	Mean	SD	*n*	Mean	SD
Age (years)		13	36.2	14.8	14	41.2	10	13	41.9	11.3
Time since diagnosis (days) (median)		12	21.5* (15.8)	21.7	14	75.6** (28.0)	19.3	13	14 (7.0)	15.1
Time to receive diagnosis (months)		12	8.8	7.6	13	18	19	12	11	10.2
			***n***			***n***			***n***	
Gender	Man		6			5			1	
	Woman		7			9			12	
Ethnicity	White		12			13			12	
	Black		0			1			1	
	Mixed ethnicity		1			0			0	
Education	Below GCSE		1			0			0	
	GCSE		4			9			3	
	A Level		3			3			2	
	Degree		3			1			6	
	Higher degree		1			1			2	
	Not known		1			0			0	
Employment status	Employed		9			12			9	
	Not employed		3			2			3	
	Voluntary		0			0			1	
	In education		1			0			0	
Type of MS	Relapsing Remitting MS		11			12			9	
	Primary Progressive MS		2			2			4	
	Secondary Progressive MS		0			0			0	

*one outlier (56 days); **two outliers (730 and 112 days); medians are provided; MS: multiple sclerosis.

Feasibility results are presented based on the Red-Amber-Green progression criteria. Table 3 summarises the Red-Amber-Green ratings. Key themes with illustrative quotations are presented in Supplementary Material 4.

**Table 3.** Red/Amber/Green ratings and suggested improvements for progression to a definitive trial, by feasibility area.

Progression criteria^2^		Rating	Suggested changes
**Recruitment**	Number of referrals	Amber	Longer recruitment period; additional sites
	Number of eligible patients, patient willingness to be recruited	Green	
**Randomisation**	Number of participants randomised	Green	Thoroughly explain randomisation process at enrolment to convey clinical equipoise
	Patient willingness		
**Attrition**	Number of participants received allocated intervention	Green	Clarify likelihood of remote delivery of support; consider recruiting peer volunteers from wider area
**Appropriateness of measures**	Completion rates, number of missing questionnaires	Amber	Acquire paper and online licences for all measures
	Acceptability	Amber	Consider alternative to Multiple Sclerosis Self-Efficacy Scale
**Nurse-specialist support intervention**	Feasibility of delivery	Red	Consider employing Assistant Psychologists to deliver the intervention to ensure sessions focus on emotional support needs and do not prioritize medical care (unrelated to emotional support).
	Acceptability	Amber	
	Intervention fidelity	Amber	
**Peer support intervention**	Feasibility of delivery	Amber	Engage peer volunteers from wider geographical region
	Acceptability	Green	
	Intervention fidelity	Green	
**Exploration of efficacy**	Signal of efficacy	Amber	Consider further training package, and/or employing Assistant Psychologists to prevent sessions prioritising medical care
	Improvers and non-improvers	Amber	

### Feasibility and Acceptability of Trial Procedures

Recruitment: We did not recruit our target sample (n = 60). A lower rate of diagnosis (based on initial clinical input) during COVID-19 partly explains our failure to recruit our target number. Patient-participant interviews suggested that the perceived appropriateness of being approached about the study by a member of the clinical team (i.e., during the diagnosis process) was influenced by whether people were expecting to receive a diagnosis of multiple sclerosis or not. Where a diagnosis was unexpected, patients felt this was ‘too soon’ because they needed time to come to terms with the shock of the diagnosis. Others felt that timing was appropriate.

Randomisation: Most found the randomisation protocol acceptable, although some felt that Interventions 1 and 2 were ‘better’ than Control.

### Appropriateness of Measures and Feasibility of Self-Report Data Collection

We had 36 (90%) and 38 (95%) questionnaire returns at 3- and 6-months follow-up, respectively; however, completion rates for individual measures ranged between 32 to 36 (80–90%), with the lowest completion rate for the EQ-5D-5L. Participants reported the questionnaire completion time was acceptable and they liked having a choice between online or paper format. They considered the Multiple Sclerosis Self-Efficacy Scale (MSSE)^12^ most difficult to complete (35; 88% completion rate) because they were uncertain how to answer some questions. Although, overall, participants felt that the questionnaires captured the most important aspects of their experiences, some thought questions were more relevant for those ‘further along’ in the disease progression.

### Feasibility of Delivering the Intervention

Following randomisation, all Intervention 1 participants received the intervention and 11 (87%) received Intervention 2. Table 4 details progression through the trial and clinical care pathway during the diagnosis process. There were eight participants undergoing diagnosis when referred to the study; seven had had their diagnosis confirmed when they consented and completed the baseline questionnaires, but *all* had a confirmed multiple sclerosis diagnosis *before* they received the intervention.

**Table 4.** Progression through the patient care and trial pathway.

Time in days	*n*	Mean (SD)	Range	Planned timescale (protocol)	Proportion of participants who met protocol planned targets
Time from point of diagnosis to first contact with research team	36^a^	11.8 (23.7)	0–112	-	-
Time from point of diagnosis to enrolment in study (consent and baseline completion)	38^b^	19.3 (24.4)	1–123	-	-
Time from point of diagnosis to MS Nurse session (Usual care)^c^	12^d^	46.3 (47.7)^e^	13–172	42	9 (75%)
Time from point of diagnosis to MS Nurse session (MS Nurse support 1)	14	39.7 (21.1)	13–79	14	2 (14%)
Time from point of diagnosis to MS Nurse session (MS Nurse + Peer support 2)	12	57.8 (36.1)	5–119	14	2 (17%)
Time from point of diagnosis to Peer support session (MS Nurse + Peer support 2)	11	83.9 (35.5)	33–134	42	2 (18%)
Time from MS Nurse support session to Peer support session (MS Nurse + Peer support 2)	11	22.1 (22.0)	3–65	28	9 (82%)

a Four participants missing as date of diagnosis was after referral. ^b^Two participants missing as date of diagnosis was after baseline measure completion. ^c^For the two-year period before PrEliMS, mean time from point of diagnosis to nurse-specialist appointment was 28 days (SD 23.5), range 0–66 days. ^d^One participant is missing as they had not been seen in any nurse-led clinic. ^e^Two outliers, 113 and 172 days, skewed the mean. MS: multiple sclerosis.

Participants in the usual care group met with a nurse-specialist within 6 weeks of their diagnosis. Due to service pressures, 22 (85%) of participants in Intervention 1 and 2 did not meet with a nurse-specialist within 2 weeks of receiving a diagnosis (as stipulated in the PrEliMS programme); however, 9 (82%) participants in Intervention 2 had their first session with a peer support worker within a month following their session with a nurse-specialist (as planned).

All 26 participants who received nurse-specialist support had one session with a nurse-specialist. Eighteen peer support worker sessions took place: with participants receiving one (n = 6), two (n = 3), or three sessions (n = 2). Pre-COVID-19, most nurse-specialist and peer support worker sessions occurred face-to-face (21 (81%) and 8 (44%), respectively); the rest occurred via telephone during the pandemic. The nurse-specialist sessions lasted on average 50 min (range 20–80 min) and the peer support worker sessions lasted on average 68 min (range 10–120 min).

### Intervention Fidelity

Content of sessions: Eighty-five per cent of nurse-specialist session record forms were completed. Most frequent topics were signposting, information provision, and symptom management. Time-sampling of 10 audio-recorded sessions showed that 55% of time was spent discussing the PrEliMS intervention content (i.e., patient-cued discussions based on the needs assessment). This included providing emotional support (references to the workbook, discussing referral to GP/psychology services) and identifying patient needs (17%). People with multiple sclerosis considered the nurse-specialists as trustworthy sources of information. However, many felt that nurses needed to focus *less* on medications, and suggested more discussion of the workbook content and emotional needs was needed.

Based on session record forms, the most common topics discussed during peer support worker sessions were signposting (18 (100% of) sessions), listening (14 sessions; 78%), and information provision (13 sessions; 72%). Indeed, both people with multiple sclerosis and nurse-specialists considered signposting, e.g., to relevant support groups, a key function of peer support. Peer support workers reported that some people with multiple sclerosis continued to attend the local support groups after the intervention ended.

Delivery of nurse specialist support: Assessment of how nurse-specialists delivered support sessions (n = 10 recordings) showed that they mostly reviewed the needs assessment document with the patient-participant (6; 60%); provided additional information on emotional needs (5; 50%); discussed the intervention's underpinning processes/model (e.g., openness to difficult experiences) (5; 50%); and were suitably flexible and responsive to issues raised (8; 80%). Total fidelity scores were between 26% and 69%, with half the sessions scored above 60% (see Supplementary Material 5).

All nurse-specialists interviewed found the session record forms beneficial because they provided structure to sessions. The quality of the sessions improved as they became more experienced, but they felt that receiving more training on psychological concepts/adjustment to diagnosis would further help them.

### Health Economics

The costs associated with Interventions 1 and 2 were estimated at £92 and £308 per participant, respectively. The most frequent resource use reported across the three groups was primary/community care, multiple sclerosis clinic, and therapy services. The key drivers of resource use at follow-up were home adaptations and hospital stays, which differed across the groups. Some issues with clarity of wording of items in the service use questionnaire were reported.

### Exploration of Efficacy

As a feasibility trial, we only explored the signal of efficacy here. Measures at baseline and results from the intention-to-treat analysis are presented in Table 5. There were no statistically significant differences between groups at 3- or 6-months follow-up for all measures, with small effect-size estimates^24^ (between 0.005 and 0.086) indicating that group-allocation accounted for less than 9% of the variance in outcomes. Individual-level reliable changes by group allocation at 3- and 6-months follow-up are summarised in Table 5 and Supplementary Material 6.

**Table 5.** Descriptive statistics of outcome measures and effect sizes by group allocation (one-way ANOVA, between group differences).

Measure	Time point	Usual care		Intervention 1 Nurse-specialist support		Intervention 2 Nurse-specialist and Peer support					Effect size
		***n***	**Mean (SD)**	***n***	**Mean (SD)**	***n***	**Mean (SD)**	**df**	***F***	***p***	**η^2^_p_ [95% CI]** ^c^
Perceived Stress Scale^a^ *Score range 0 to 16*	Baseline	13	7.38 (3.1)	14	6.79 (3.6)	13	7.08 (2.7)	2, 39	0.121	0.887	
	3 months	13	5.69 (3.6)	13	6.00 (3.6)	12	6.67 (4.3)	2, 37	0.218	0.805	0.012 [0–0.10]
	6 months	13	6.77 (3.6)	13	6.31 (3.8)	13	6.31 (3.8)	2, 35	0.092	0.912	0.005 [0–0.06]
HADS Anxiety Scale^a^ *Score range 0 to 21*	Baseline	13	9.00 (4.6)	14	7.93 (4.1)	13	10.54 (4.6)	2, 39	1.181	0.318	
	3 months	13	7.08 (4.0)	13	7.54 (3.9)	12	9.33 (6.1)	2, 37	0.784	0.464	0.041 [0–0.18]
	6 months	13	7.38 (4.4)	13	7.77 (4.1)	10	9.50 (5.1)	2, 35	0.686	0.511	0.038 [0–0.18]
HADS Depression Scale^a^ *Score range 0 to 21*	Baseline	13	6.31 (5.4)	14	4.71 (3.9)	13	7.69 (4.1)	2, 39	1.499	0.237	
	3 months	13	5.85 (6.0)	12	5.83 (5.1)	12	7.83 (5.3)	2, 36	0.531	0.593	0.029 [0–0.16]
	6 months	13	5.85 (5.2)	13	6.15 (4.7)	10	7.60 (4.6)	2, 35	0.403	0.671	0.023 [0–0.14]
Multiple Sclerosis Impact Scale – Psychological Sub-Scale^a^ *Score range 9 to 36*	Baseline	13	23.85 (6.6)	13	21.54 (5.3)	13	23.69 (7.5)	2, 38	0.507	0.606	
	3 months	13	20.38 (7.1)	13	20.23 (7.0)	12	21.92 (8.0)	2, 37	0.198	0.821	0.011 [0–0.10]
	6 months	13	20.69 (6.9)	13	21.85 (7.5)	10	23.20 (7.8)	2, 35	0.327	0.724	0.018 [0–0.13]
Multiple Sclerosis Self-Efficacy Scale^b^ *Score range 14–84*	Baseline	10	50.40 (10.7)	14	53.86 (14.2)	13	46.23 (14.9)	2, 36	1.057	0.359	
	3 months	13	53.77 (13.0)	13	49.54 (14.3)	12	47.67 (13.2)	2, 37	0.674	0.516	0.035 [0–0.17]
	6 months	13	51.46 (17.2)	12	50.58 (13.4)	10	47.70 (12.5)	2, 34	0.195	0.824	0.011 [0–0.10]
EQ-5D-5L Visual Analogue Scale^b^ *Score range 0 to 100*	Baseline	13	75.00 (15.0)	12	69.92 (22.2)	12	61.25 (26.8)	2, 36	1.273	0.293	
	3 months	12	69.29 (28.0)	10	72.50 (20.6)	11	67.73 (29.4)	2, 32	0.088	0.916	0.005 [0–0.07]
	6 months	13	76.92 (20.0)	12	62.08 (23.8)	7	70.71 (21.5)	2, 31	1.452	0.251	0.086 [0–0.26]

a Higher scores indicate greater stress, anxiety and depression. ^b^Higher scores indicate greater self-efficacy and health; ^c^*n^2^_p_* effect size index^1^: 0.01 small effect, 0.06 medium effect, 0.14 large effect. The EuroQol 5 Dimension 5 Level (EQ-5D-5L); Hospital Anxiety and Depression Scale (HADS); Multiple Sclerosis (MS); Multiple Sclerosis Impact Scale-29 (MSIS-29)–psychological subscale (MSIS-psy); Multiple Sclerosis Self-Efficacy Scale (MSSE); Perceived Stress Scale 4-item (PSS-4).

Power and sample size calculations, based on minimal clinically important difference (at 6-months follow-up) are presented in Table 6. Taking attrition into account, the sample size in a definitive trial would be between 162 and 186 participants, depending on the primary outcome measure chosen.

**Table 6.** Sample size calculations based on minimal clinically important difference* (based on 6 months follow-up).

Measure	Minimal clinically important difference*	Sample size per group	Sample size per group with attrition	Total (2 groups)
Perceived stress scale	1.73 (0.5SD) points	78	87	174
MSIS-Psy	3-point difference (suggested); in PrEliMS sample 3.62 (0.5SD) points	78	87	174
MS Self-Efficacy scale (MSSE)	7.19 (0.5SD) points	78	89	178
HADS anxiety	Published cut-off 10 points; in PrEliMS sample 2.23 (0.5SD) points	73	81	162
HADS depression	Published cut-off 10 points; in PrEliMS 2.39 (0.5SD) points	84	93	186

Significance level (alpha) of 2.5%, power (i-beta) of 80%, two-tailed, calculated via G*Power; 0.5SD change considered clinically meaningful if there is no published minimal clinically important difference.^25^ Hospital Anxiety and Depression Scale (HADS); Multiple Sclerosis (MS); Multiple Sclerosis Impact Scale-29 (MSIS-29)–psychological subscale (MSIS-psy); Multiple Sclerosis Self-Efficacy Scale (MSSE); Perceived Stress Scale 4-item (PSS-4).

## Discussion

Overall, it appears it is feasible to conduct a definitive trial, the PrEliMS interventions are acceptable, and patients request such support. However, some changes to the design are required before this intervention is taken forward.

In terms of recruitment, the number of referrals (three per month) was lower than anticipated, but consistent with the diagnosis rate at multiple sclerosis clinics and is consistent with similar studies.^26^ A longer recruitment period and/or additional study sites would improve recruitment rate. Of those approached, 67% met the eligibility criteria, were willing to be recruited, and consented to participate.

A strong preference for a particular treatment group, and differences in the acceptance of clinical equipoise determine whether patients agree to be randomised.^27^ Although patients perceived the intervention groups as ‘better’ than control, all agreed to be randomised and none discontinued due to their group allocation. However, this may raise expectancy bias because we cannot blind participants to treatment allocation.^28^ Therefore, clinical equipoise could be more clearly explained during the randomisation process.

Attrition was low across groups. Two participants did not receive the intervention (due to logistical and contact issues) in Intervention 2. Although only relevant to one participant, this highlighted potential challenges in organising peer support worker sessions if individuals live long distances from each other or are reluctant to receive support remotely (telephone or online). We may need to recruit more peer support workers from different regions and participants understand that sessions could be remotely-delivered.

Overall, outcome measure completion rates across all groups and time points were high. Generally, participants found the questionnaires easy and quick to complete. However, participants found the MSSE difficult, and the number of missing items suggest that this might need to be reconsidered for a definitive trial. There were also several missing EQ-5D-5L questionnaires, partly because our licence only included paper copies. Obtaining electronic versions of the EQ-5D-5L license may remedy this.

Although the nurse-specialist support intervention was delivered within the recommended NICE^3^ timelines (of first appointment occurring within 6 weeks of diagnosis), only a small proportion of sessions occurred within our planned 2 weeks following diagnosis. The PrEliMS interventions were co-designed with key stakeholders^7^ (people with multiple sclerosis, carers/family, healthcare professionals, including nurse-specialists) who jointly agreed that the optimal time for the first nurse-specialist appointment was 2 weeks post-diagnosis, but also acknowledged that the timing of the intervention depended on patients’ needs and preferences. We found that it was not feasible to deliver the intervention as per the stakeholders’ suggested timeframes because of nurse staffing constraints. Therefore, a more flexible person-centred approach is required.

Participants perceived the support provided by nurse-specialists as trustworthy and credible, but felt that the primary focus should not be on medication alone. Indeed, they felt that psychological aspects and how to obtain support and further information to be lacking from their initial diagnostic consultations.^29^ As it may not be possible for nurse-specialists to deliver the intervention in their current workplan (due to capacity issues, experience of nurse-specialists with psychological aspects of multiple sclerosis), we suggest that another workforce (e.g., assistant psychologists) may be better placed to deliver the intervention.

Peer support was positively received. The key feasibility issue was needing more peer support workers from diverse locations to enable more in-person sessions if requested by people with multiple sclerosis.

As a feasibility trial, the study was not powered to detect between-group differences, therefore analyses only offer trends in the data. Exploratory analyses indicated predominantly small effect sizes between groups on all measures at both follow-up periods, consistent with the mixed findings in individual changes (see Supplementary Material 6 and 7) with no differences between the control and intervention groups. One reason could be due to contamination, in that by requesting nurse-specialists to complete session record forms, usual care may have inadvertently changed.

Furthermore, in our multiple sclerosis clinics, like in many others, with nurse-specialists’ increasingly focusing on discussing and monitoring the use of Disease Modifying Therapies,^30,31^ perhaps there is little time or resource allocated to discuss psychological issues. This is evidenced from the intervention fidelity findings and poses an implementation (including training) consideration in a definitive trial.

Although we recruited from different multiple sclerosis clinics, a limitation of this study is that people with multiple sclerosis and nurse-specialists were recruited from a single NHS centre and peer support workers from one multiple sclerosis charity, which may not therefore be representative of people accessing and delivering services elsewhere. Another limitation of is that we did not reach our recruitment target of 60 participants. However, we had set a higher recruitment target to account for possible dropouts. Furthermore, the number of participants randomised into each group met the minimum recommended sample sizes of 10 per treatment group of 10 for standardised small or medium effect sizes.^15^

Another issue is the timing of the delivery of the intervention. We recruited people who were within two years of multiple sclerosis diagnosis *or* were undergoing diagnosis process. This timeframe was chosen because (i) the diagnostic process can be lengthy and complicated, and often there is no single date of diagnosis; (ii) our Patient and Public Involvement group members felt that the adjustment period was protracted, and having a shorter period would exclude those still experiencing adjustment difficulties, and (iii) findings from our meta-review^6^ and stakeholder focus groups^7^ indicated that it was important to balance the provision of reliable sources information, with the need to allow individuals to process the diagnosis in their own time, before providing them with further support. Consequently, while we elected to be inclusive, this has created a heterogenous group, raising issues related to heterogeneity of treatment effects.^32^

In conclusion, our findings suggest that it is largely feasible to conduct a definitive trial and that the PrEliMS interventions are acceptable and patients are requesting such support. However, some changes to the design are required. As the combination of nurse-specialist and peer support was identified as providing different, but complimentary, support to those newly diagnosed, and because we did not find a signal of efficacy in this feasibility trial, we suggest that future trials test our combination intervention (i.e., Intervention 2) compared to usual care. A cluster trial design may address issues of possible contamination of usual care, but some questions remain around whether outcomes can be improved by having a dedicated workforce to deliver the intervention, which may well be within the purview of psychology and/or rehabilitation specialists. Given that the delivery of the intervention by another workforce has not been formally tested in our trial, a definitive trial may benefit from an internal pilot to assess any new issues in intervention delivery. Given the complexities in arriving at a diagnosis of multiple sclerosis and the increased pressures within clinical services, the timelines for the delivery of the intervention need to be more patient centred, flexible, and in keeping with service realities and patient needs. Based on the current NICE^3^ guidelines, providing the intervention within 6 weeks of diagnosis appears more realistic.

**Clinical messages**

- It is feasible to deliver such a programme, but it may need to be delivered by psychologists or other rehabilitation professionals.
- People with multiple sclerosis perceived the support provided by nurse-specialists as trustworthy and credible, but felt that the primary focus should not be on medication alone but should also cover emotional needs.
- Nurses may require additional support and training to address emotional and adjustment issues with people with multiple sclerosis.

## Supplemental Material

sj-docx-1-cre-10.1177_02692155241284781 - Supplemental material for Providing Emotional Support During the Process of Multiple Sclerosis Diagnosis (PrEliMS): A Feasibility Randomised Controlled Trial Supplemental material, sj-docx-1-cre-10.1177_02692155241284781 for Providing Emotional Support During the Process of Multiple Sclerosis Diagnosis (PrEliMS): A Feasibility Randomised Controlled Trial by Roshan das Nair, Jacqueline R Mhizha-Murira, Gogem Topcu, Tierney Tindall, Clare Bale, Nima Moghaddam, Grit Scheffler-Ansari, Avril Drummond, Deborah Fitzsimmons and Nikos Evangelou in Clinical Rehabilitation
