# Supplementary material for: Providing Emotional Support During the Process of Multiple Sclerosis Diagnosis (PrEliMS): A Feasibility Randomised Controlled Trial
Source: Clin Rehabil. 2024 Sep 25;38(11):1506–20. doi: 10.1177/02692155241284781 (PMC11528975; doi:10.1177/02692155241284781)
Supplement: sj-docx-1-cre-10.1177_02692155241284781 - Supplemental material for Providing Emotional Support During the Process of Multiple Sclerosis Diagnosis (PrEliMS): A Feasibility Randomised Controlled Trial [file sj-docx-1-cre-10.1177_02692155241284781.docx]

# Supplementary Materials

**Supplementary Material 1.** The PrEliMS Intervention Template for intervention and replication (TIDieR) checklist^3^

| 1.What is the **NAME** of the intervention? | An intervention to provide emotional support during the Multiple Sclerosis diagnosis process (PrEliMS) |
| --- | --- |
| 2.**WHY** do the intervention? | *Intervention rationale:*  The period surrounding MS diagnosis can be highly stressful for both the patients and their families due to long, complicated and challenging diagnosis process. Challenges faced during diagnosis may influence patients’ perceptions of MS and their relationships with the healthcare team. The way in which the diagnostic phase is managed may contribute to how successfully patients adjust to MS.  NICE and European MS Platform recommend providing accessible information, informed advice and support at diagnosis.  There is currently poor support and information provision for people with MS around the diagnosis process. |
|  | *What are the underpinning theories?*   - Model of adjustment to MS diagnosis (developed based on PrEliMS meta-synthesis findings)^4^: People with MS experience several negative emotions and external stressors around the time of diagnosis, which might limit their ability to make sense of MS diagnosis and to adjust to this new and uncertain situation. However, coping and helpful resources might help reduce the negative impact of being diagnosed and facilitate the adjustment process to MS diagnosis - PrEliMS Meta-review findings^4^:   - Factors relating to psychosocial adjustment (e.g., negative emotional responses, positive emotional responses, impact on daily life, the impact of family on adjustment, personal attributes, management techniques, and the diagnostic process).   - Models of psychosocial adjustment:   - Working model of adjustment^5^   - Biopsychosocial model   - Coping theory   - Health Psychology models of health behaviours   - Model of emotional adjustment and hope^6^   - Model of the psychological impact of the unpredictability of MS^7^   - Protection motivation model   - Social cognitive theory - Frameworks relevant to psychosocial adjustment in MS:   - European MS Platform code of practice   - International Classification of Functioning, Disability and Health framework   - NICE guidelines   - National Service Framework for long-term conditions - Processes associated with the following therapies/therapeutic interventions that served as models:   - Cognitive Behavioural Therapy   - Acceptance and Commitment Therapy   - Mindfulness   - Motivational interviewing   - Psychoeducation   - Supportive counselling/psychotherapy   - Available interventions for psychosocial adjustment: Interventions varied from cognitive behavioural approaches, relaxation activities, physical activities, educational programmes, counselling, and social support groups. There were also coping-based, self-management and symptom management interventions. - Preliminary pathway developed based on literature and PPI input for providing emotional support and advice to people around the MS diagnosis process, bridging the gap between MS clinics to MS Society - Stakeholder views and feedback (Focus groups with people with MS, carers/families, health professionals, MS Society staff and volunteers):   - Need for a point of contact to ask questions   - Tailored support based on needs (when, how and what to receive)   - Need for timely information and advice   - Early referral to MS Society   - Talking to someone with lived experience - MacMillan Cancer support – Just been diagnosed (example model) - Shift.ms (example model) - Person-based approach to intervention development |
|  | *Goals of the elements essential to the intervention?*  **MS Nurse Support:** To provide standardised support and advice to patients at diagnosis, to better cope with MS diagnosis and associated emotional demands, and improve their mood, self-efficacy and quality of life.  **Peer Support:** Opportunity for newly diagnosed patients to discuss concerns, worries and problems and share feelings and experiences with another experienced patient (patient with lived experiences) in a non-judgmental, safe environment to help patients feel listened to, experience empathy, feel more empowered about their own feelings, and also help them find the most appropriate support. |
| 3. **WHAT** **materials** were needed for the intervention? | *Training materials:*   - MS Nurse training pack - Peer Support in Long Term Conditions document^8^ |
|  | *Provider materials:*   - MS Nurse Support facilitator guide - Better Living with MS toolkit - Standardised referral letters - Peer Specialist Toolkit^9^ |
|  | *Participant materials:*   - Supportive handouts |
| 4. What **PROCEDURES** took place in the intervention? | *Recruitment of Peer Support Workers:*   - MS Society local branches - MS Patient and Public Involvement Groups |
|  | *Provider training:*   - Group training for Neurologists and MS Nurses (provided by experienced clinical psychologists) - On-going supervision (provided by experienced clinical psychologists) and peer support for MS Nurses - Group training for Peer Support Workers (provided by experienced clinical psychologists) - Supervision training to MS nurses (provided by experienced clinical psychologists) |
|  | *Recruitment of patients:*  MS Clinics |
|  | *Intervention:*   - MS Nurse support:   - Face-to-face/phone session with MS Nurse – included answering patients’ questions about MS, identifying support needs, providing psychoeducation (e.g., teaching adaptive coping strategies), teaching acceptance and commitment strategies^10^, and referring to other services (based on needs)   - (for ‘Peer Support’ intervention component only) Triaging to a Peer Support Worker - Peer Support: Face-to-face//phone/email session with a Peer Support Worker (a patient/carer with lived experience) – included supportive listening and following the Mental Health Foundation’s “Peer Support in Long Term Conditions’ document^8^ and the “Peer Specialist Toolkit” developed by the Veterans Integrated Service Network (VISN) 1 New England of Mental Illness Research Education and Clinical Centre (MIRECC)-Peer Education Centre, and the VISN 4 MIRECC-Resource Centre^9^. |
|  | *Intervention structure:*   - MS Nurse Support: One face-to-face (or telephone during COVID-19 restrictions) session with MS Nurse within an average of 7 weeks of diagnosis with telephone contact as needed. Sessions lasted an average of 50 minutes (range 20-80 minutes). - Peer Support: Patients were triaged to a Peer Support Worker within one month following their session with the MS Nurse. Most participants received one face-to-face/phone session with the Peer Support Worker, scheduled at a convenient time (and location, where applicable) for both the Peer Support Worker and the patient. The sessions lasted an average of 60 minutes (range 5-120 minutes). |
| 5. **WHO** was involved in the intervention? | *Intervention providers:*   - MS Nurse support provided by Specialist MS Nurses who were trained to deliver the intervention - Peer Support provided by ‘Peer Support Workers’ who are patients/carers with lived experience and who were trained to deliver the intervention |
|  | *Participants:*   - Newly diagnosed MS patients (any type) and who were aged 18 years or over. |
| 6. **HOW** was the intervention delivered? | - ‘MS Nurse Support’ intervention component delivered one-to-one, face-to-face/phone.   - Phone call by the MS Nurse to supplement the sessions (instigated by patient based on needs) - ‘Peer Support’ intervention component delivered one-to-one: either face-to-face or over the phone or via email (follow-up sessions), based on patient preference. - Programme structure, i.e., number of sessions and frequency, was tailored to the patient based on preference and need. - Carers or family members were able to attend the sessions. - Intervention could be paused based on patient needs and can be placed on a waiting list until the patient is ready to engage. - Goal-setting was used to tailor the intervention to needs of participants |
| 7. **WHERE** was the intervention delivered? | - MS Nurse Support: Hospital (e.g., MS Clinics) - Peer Support: Community (e.g., university, café, park), over the phone or via email (follow-up sessions). |
| 8. **WHEN** was the intervention delivered? **HOW MUCH**? | - MS Nurse Support: Appointment with an MS Nurse within 7 weeks (an average of 50 minutes). Peer support: Meeting with a Peer Support Worker 4 weeks of diagnosis (minimum of one session – each session an average of 60 minutes). |
| 9. How was the intervention **TAILORED**? | The intervention was tailored according to needs, abilities, and comorbidities of the individual patient.  Patients were asked to think about 5 top priority needs before they attended the MS Nurse session and to bring this list with them to tailor the information/support provided accordingly. |
|  | *Tailoring occurred through:*   - Content (information on MS and services, coping strategies, support) - Delivery (day, time, number of sessions, who delivers sessions [for Peer Support component only]) - Goal-setting (needs, aims) - Progression (readiness to connect to MS Society) |
|  | *Tailoring depended on:*   - Information needs: type of information on MS and services, amount of information - Emotional needs: willingness/readiness to talk to a patient/carer Support Worker, identifying mood problems – referral pathway - Social support: level of involvement of family members/carers - Comorbidities and physical/cognitive symptoms of patients - Environmental context |
| 10. **MODIFICATIONS** to the intervention | *What, why, when and how:*  The intervention was not modified during the course of the study. |
| 11. **HOW WELL? PLANNED** (How the intervention adherence and fidelity was assessed?) | *How and by whom:*  We requested MS Nurses and patients consent to audio-record sessions. Furthermore, MS Nurses and Peer Support Workers were requested to complete record forms detailing topics discussed during the sessions (including information provided). |
|  | *Strategies used to maintain/improve fidelity:*  These recorded sessions were compared to the intervention manual. We mapped the data onto the key elements of content, coverage, frequency and duration, paying attention to facilitation strategies, quality of the delivery and participant responsiveness^11^ |
| 12. **HOW WELL?**  **ACTUAL** (the extent to which the intervention was delivered as planned) | - MS Nurse support: Most of the MS Nurse support sessions took place within 7 weeks following diagnosis (planned timescale 2 weeks). During the support sessions only 55% of the time was spent discussing the PrEliMS intervention content (including workbook and emotional needs). - Peer Support: Most of the Peer Support sessions occurred almost 12 weeks following diagnosis (planned timescale 6 weeks). The topics discussed during the sessions as detailed on the record form were consistent with the goals (i.e., content) of the intervention. The minimum number of sessions that were *actually* delivered (one) were lower than those planned (minimum of two sessions). |

MS: multiple sclerosis

**Supplementary Material 2a.** Semi-structured interview guide: Patient participants

*[Please note: This is a semi-structured topic guide that is designed to be used flexibly with each participant. As such, the questions and prompts (presented as sub-questions) asked in each interview are likely to vary slightly.]*

Opening question

1. Please can you tell me about your experience of being involved in the study?

Recruitment and group allocation

1. How did you come to be involved in the study?
   1. What did that feel like?
2. How did it feel to be allocated to your study group?
   1. How did it feel (not) to be allocated to receive the intervention (extra support from the Nurses and support workers)?

Study procedures

1. What did you think about the information we collected from you at the beginning and end of the study?
   1. How easy (or not) were the questionnaires to complete?
   2. What did you think about how many questionnaires you needed to complete?
   3. Did the questionnaires ask about things that were relevant for you, in relation to what the study was about?
   4. How would you rate the assessments on a scale of 1-10 (1 did not capture important aspects of my experience to 10 fully captured the important aspects of my experience)

**For intervention participants only**

1. How did you find the intervention (support programme)?
   1. What did you find helpful about the MS Nurse Support sessions? Any particular aspects?
   2. [Ask if in Peer Support Group] What did you find helpful about the Peer Support sessions? Any particular aspects?
   3. What did you find unhelpful about the support programme [Enhance MS Nurse Support / Peer Support sessions]? Any particular aspects?
   4. Were there any particular aspects which were good or bad?
2. How do you think we could improve the support programme in the future?
3. Would you recommend this support programme to other newly diagnosed people with multiple sclerosis?

Impact/Perceived benefits

1. Have you experienced any changes since taking part in this study?
   1. What are these changes?
   2. How do you make sense of these changes?

Other issues

1. Is there anything else you would like to tell me about?

**Supplementary Material 2b.** Semi-structured interview guide: Intervention Providers

*[Please note: This is a semi-structured topic guide that is designed to be used flexibly with each participant. As such, the questions and prompts (presented as sub-questions) asked in each interview are likely to vary slightly.]*

Opening question

1. Please can you tell me about your experience of being involved in the study?

Intervention delivery

1. Please can you tell me about your experiences of delivering the ‘Enhanced MS Nurse Support’ / ‘Peer Support’ [Ask as appropriate]?
   1. How easy/difficult was it to implement the support programme with newly diagnosed people with MS?
      1. MS nurses – ask them whether they think they are best placed to deliver the intervention. If not, who then?
   2. What went well?
   3. What were the difficulties and how did you overcome them?
   4. How did you find the training that you received?
   5. How did you find using the manual?
   6. How did you find the clinical supervision at site/by the trial therapists?
   7. How did you find the monitoring of your practice by the research study? What was it like using the workbook?
   8. How does the intervention compare to usual care? Is it the same/different?
   9. What are the possible reasons why there was such variation when participants were seen by the nurses (aim was for sessions to occur within 2 weeks of diagnosis? Some participants were not seen until over 12 weeks later)
2. How did participants find the support programme?
   1. Were there any particular aspects which were good or bad?
   2. Did participants experience any changes/benefits from the support programme?
3. How do you think the support programme could be improved in the future?
4. Would you recommend this support programme to other MS Nurses / Peer Support Workers [ask as appropriate] working with newly diagnosed people with MS?

Study procedures

1. Please can you tell me about the recruitment process?
   1. How could this be improved for a future trial?
2. How did you find the study procedures, i.e., working to the protocol?
   1. How could this be improved for a future trial?
3. What did you think about the measures we used at baseline and follow-ups?
   1. How would you rate the assessments on a scale of 1-10 (1 did not capture important aspects of the experience to 10 fully captured the important aspects of the experience)

Service barriers and facilitators **[For MS Nurses only]**

1. Please can you tell me about your experience of working on the trial within your department?
2. What are the main barriers to integrating the trial practice into the wider service of your department?
3. What are the main facilitators of integrating the trial practice into the wider service of your department?

Barriers and Facilitators **[For Peer Support Workers only]**

1. Please can you tell me about your experience of doing the sessions in community settings or online (via Skype)
2. What are the main barriers to deliver the peer support sessions in community settings / online?
3. What are the main facilitators to deliver the peer support sessions in community settings / online?

Other issues

1. Is there anything else you would like to tell me about?

**Supplementary Material 3.** Study objectives, outcome measures, data collection points and data sources

|  | **Objective** | **Data/Outcome measure** | **Analysis** | **Data sources** |
| --- | --- | --- | --- | --- |
| **1** | **Feasibility of proceeding to a Phase III trial** | | | |
|  | 1. Feasibility of trial procedures | Feedback interview data from participants and service providers (i.e., questions about the research process/procedures, and suggested changes to the study) | Framework analysis | Semi-structured interview transcripts.  Patient-participants: between 3- and 6-month follow-up.  Service providers: after the end of recruitment. |
|  | 1. Acceptability of trial procedures | Feedback interview data on the trial procedures | Framework analysis | As above. |
|  | 1. Feasibility of randomisation protocol | Feedback interview data on randomisation protocol and willingness and acceptance of patients to be randomised | Framework analysis | As above. |
|  | 1. Feasibility of recruitment | Number of patients newly diagnosed with MS during the period of recruitment and referred to the clinical team | Frequencies / percentages | Referral, screening and recruitment logs. CONSORT diagram. |
|  |  | Number of patients who met the eligibility criteria | Frequencies / percentages | As above. |
|  |  | Number of consenting/randomised patients | Frequencies / percentages | As above. |
|  |  | Reasons for non-participation | Frequencies / percentages | As above. |
|  |  | Retention rates | Frequencies / percentages | As above |
|  |  | Feedback interview data – participant views of recruitment | Framework analysis | Patient-participant semi-structured interviews transcripts. |
|  | 1. Estimating sample size needed for a Phase III RCT | Effect sizes from ANOVAs, standard deviations and attrition rates | Sample size calculation | Participant questionnaires, with data inputted into outcomes database (excel). |
|  | 1. Appropriateness of measures | Completion rates of outcome measures | Percentage | Participant log. Study CONSORT diagram. |
|  |  | Number of missing online and postal data | Percentage | As above |
|  |  | Estimates of time (minutes) taken to complete measures (from online/phone data and feedback interviews) | Descriptive statistics | Participant log. |
|  |  | Feedback interview data – participant views of the appropriateness of measures | Framework analysis | Patient-participant semi-structured interview transcripts |
|  | 1. Feasibility of self-report data collection | Number of missing online and postal data | Frequencies / percentages + partial breakdown by items (areas of difficulty?) | Participant log. |
|  | 1. Feasibility of audio recording support sessions | Number of participants consenting to audio recording | Frequencies / percentages | Consent form and Participant log. |
|  |  | Number of support sessions audio recorded | Frequencies / percentages | As above. |
|  |  | Feedback interviews – participant views of audio recording support sessions | Framework analysis | Patient-participant and MS nurse semi-structured interview transcripts. |
| **2** | **Feasibility of the MS diagnosis emotional support intervention** | | | |
|  | 1. Acceptability of intervention (Support 1 and Support 2) | Drop-out rate (and reasons for withdrawal) | Frequencies / percentages | Participant log. |
|  |  | Number of nurse support & peer support sessions completed | Descriptive statistics / average percentage completion (and range) | As above. |
|  |  | Feedback interview data | Framework analysis | Patient-participant and MS nurse semi-structured interview transcripts |
|  | 1. Feasibility of delivering Support 1 (MS nurse) intervention | Operational issues in delivering intervention through feedback interview data | Framework analysis | MS nurse semi-structured interview transcripts. |
|  |  | Missed and rescheduled support sessions | Percentage | Participant log |
|  |  | Length of sessions (minutes) | Average (SD) | Session audio recordings |
|  | 1. Feasibility of delivering Support 2 (peer support) intervention | Operational issues in delivering intervention through feedback interview data | Framework analysis | MS nurse and peer support worker semi-structured interview transcripts. |
|  |  | Missed and rescheduled support sessions | Percentage | Participant log. |
|  |  | Length of sessions | Average (SD) | Peer support worker session record forms. |
|  | 1. Credibility of intervention | Feedback interview data | Framework analysis | Patient-participant, MS nurse and peer support worker semi-structured interview transcripts |
|  | 1. Fidelity of intervention | Sample of support sessions audio data | Percentage | Session audio recordings. |
|  |  | Content of sessions as reported on the MS nurse and Peer support worker record forms | Fidelity rating against criteria for PrEliMS model consistency - Time-sampling, based on minute-by-minute coding of content, and saliency analysis of intervention transcripts | Nurse support session record form. |
|  | 1. Documentation of usual care further | Using service use questionnaire | Frequencies / percentages / descriptive statistics | Participant questionnaires, with data inputted into outcomes database (excel). |
|  |  | Feedback interview data | Framework analysis | Patient-participant and MS nurse semi-structured interview transcripts |
|  | 1. Feasibility of collecting data for an economic evaluation using a bespoke service use questionnaire | Number of missing or clearly invalid service use questionnaire (SUQ) data | Frequencies / percentages + partial breakdown by items (areas of difficulty?) | Participant questionnaires, with data inputted into outcomes database (excel). |
|  |  | Completion rates | Frequencies / percentages / descriptive statistics | As above. |
|  |  | Exploration of possible ceiling effects | Descriptive statistics | As above. |
|  |  | Feedback interview data | Framework analysis | Patient-participant semi-structured interview transcripts |
| **3** | **Other outcomes** | | | |
|  | 1. Perceived stress | PSS4 (Baseline and follow-up [3 & 6 months]) | Individual changes – Reliable Change Index (RCI) and Clinically Significant Change (CSC) | Participant questionnaires, with data inputted into outcomes database (excel). |
|  | 1. Mood | HADS (Baseline and follow-up [3 & 6 months]) | Individual changes – Reliable Change Index (RCI) and Clinically Significant Change (CSC) | As above. |
|  | 1. Psychological impact of MS | MSIS-29 – psychological subscale (Baseline and follow-up [3 & 6 months]) | Individual changes – Reliable Change Index (RCI) and Clinically Significant Change (CSC) | As above. |
|  | 1. Self-efficacy | MSSE (Baseline and follow-up [3 & 6 months]) | Individual changes – Reliable Change Index (RCI) and Clinically Significant Change (CSC) | As above. |
|  | 1. Health-related quality of life | EQ-5D-5L (Baseline and follow-up [3 & 6 months]) | Individual changes – Reliable Change Index (RCI) and Clinically Significant Change (CSC) | As above. |
|  | 1. Exploration of effectiveness | PSS4, HADS, MSIS, MSSE | ANOVA | As above. |
|  | 1. Exploration of resource use and costs | Service use questionnaire alongside resource use and costs of PrEliMS vs usual care | Descriptive analysis of resource use and costs based on available cases. | As above. |

**Supplementary Material 4.** Patient-participant, nurse-specialist and peer support worker feedback interviews: key themes and subthemes and illustrative examples

| **Theme** | **Sub-theme** | **Illustrative quotes** |
| --- | --- | --- |
| Research processes | Recruitment | 1. When he [neurologist] said about the study, I think that was good because I feel it’s always good to participate in things that are going to improve the way a condition is handled or maybe just provide that extra bit of information. (Woman, aged 51-60, 4 days since diagnosis, Usual Care group)  2. I can't remember exactly when I was asked to join the study, so possibly it weren't the right time […] it’s not a great time” (Man, aged 51-60, 1 week since diagnosis, MS Nurse support group)  3. I didn’t think it [being introduced to the study during the diagnosis process] was too bad […] I think I also didn’t take the news as bad as other people would. (Male, aged 21-30, 6 weeks since diagnosis, Usual Care group)  4. I think either way, it [recruitment] should be done within a short space of time […] if you hear about it [study] you can start processing things, but if you hear about it today or two months later, you're going to try and start having another conversation and rehashing all of that information. (Female, aged 41-50, 3 weeks since diagnosis, Intervention 2 group) |
|  | Randomisation | 1. Obviously I was devastated by getting the news [diagnosis], so to be on the study I felt I was going to get the maximum help I could get, which really did make me feel more positive. (Female, aged 51-60, 1 day since diagnosis, Intervention 1 group)  2. [I] certainly think that you definitely [...] want to be in [at] least my group. (Male, aged 51-60, 1 week since diagnosis, Intervention 1 group)  3. I did want the third group [Intervention 2], to be honest, because with being totally ignorant to MS, it would have been good to have the full support and indeed the support group (Male, aged 41-50, 1 month since diagnosis, Intervention 1 group)  4. I felt fine, no feelings either way really, I kind of knew that by taking part you're agreeing to, you know, the terms of the study and that if you're not happy I can change my mind later. I felt fine, no feelings either way really, especially because there’s no change to the care I would have received anyway. (Female, aged 31-40, 1 week since diagnosis, Usual Care group) |
| Study questionnaires | Appropriateness of measures – relevance of content | 1. I did think they [questionnaires] were relevant, whether or not it happened to me or not, but yeah, I would think so. (Female, aged 31-40, 2 months since diagnosis, Intervention 2 group)  2. I think the questions were quite broad enough in terms of physical wellbeing and mental wellbeing and, you know, support and things like that, but I think, I remember thinking at the time yeah there’s quite a few questions but you can understand why if you were trying to get a holistic picture of how people are doing post diagnosis, because all of those things factor into that, so yeah I can understand why the questions were as they were (Female, aged 31-40, 1 week since diagnosis, Usual Care group)  3. The questionnaires ask things about you know, my day to day activities aren’t affected in the slightest. I don’t need anybody to help me do anything. I don’t see the consultant and the MS nurse regularly. I haven’t been put on any medication yet. I was diagnosed in August […] I didn’t feel like you probably get much out of my questionnaire because a lot of it was like yeah, I’m fine. No that doesn’t apply. (Female, aged 31-40, undergoing diagnosis, Intervention 1 group) |
|  | Feasibility of data collection - format, and ease of completion | 1. The questionnaires were, I’d say, exactly how I’d imagine them. It takes me about 15 minutes to do each one (Male, aged 21-30, 3 weeks since diagnosis, Usual Care group)  2. The amount of questionnaires was fine (Female, aged 41-50, 4 weeks since diagnosis, Intervention 1 group)  3. A couple of times I didn’t know whether to answer yes or no. I may have got it [MSSE questionnaire] wrong, the wrong way round. (Female, aged 60+, 3 weeks since diagnosis, Intervention 2 group)  4. I’ve filled the questions [MSSE questionnaire] out, but then I think to myself I’ve scored that the wrong way round, should have been on the other way. (Female, aged 41-50, 4 weeks since diagnosis, Intervention 1 group) |
| MS Nurse Support | Feasibility of delivery - timing of the sessions | 1. Yeah, but it would have been nice perhaps to have somebody say to me, like, this is going to be a bit of a shock, we’re going to, you know, in a couple of days you'll see an MS nurse and to suggest that I did write down any problems that I was having so that you had someone to talk to them, you know, a bit more structure to talk to them about. (Female, aged 51-60, 1 day since diagnosis, Intervention 2 group)  2. *Interviewer*: So, when do you think you would have benefited from seeing the MS nurse after your diagnosis, at which point do you think would it have been best for you? Interviewee: I think week after. I mean, I don't know if that’s too early, but I think you need to be in there straightaway with it, to be honest, I really do, because it’s the most frightening period. (Female, aged 41-50, 4 weeks since diagnosis, Intervention1 group)  3. *Interviewer*: So, our plan was to recruit newly diagnosed patients, and then within 2 weeks they receive PrEliMS. How has that worked in practice? *MS Nurse*: So, in practice, not so well, because the nurse capacity has been a problem recently, and we haven’t been able to fulfil that at all [,,,] If in the event we see them [patients] for newly diagnosed counselling, we often do see them quite soon after to discuss the treatment anyway. So, it sort of happens. *Interviewer*: So practically speaking, would you say it’s realistic or not to have it [first MS Nurse Support session] within those first 2 weeks, take into account the capacity issues. *MS Nurse*: No, not at the moment. Interviewer: What would be more feasible then? *MS Nurse*: Within 6 weeks. |
|  | Content of sessions | 1.Interviewer: What was particularly helpful about that session with the MS nurse? Interviewee: I think it was the sort of information and support and the fact I know if something was happening, who to call and I know where to go to. (Female, aged 21-30, 6 days since diagnosis, Intervention 2 group)  2. The thing that I found helpful, or the best thing, I suppose was a little bit better understanding of what the condition [multiple sclerosis) was. (Male, aged 51-60, 1 week since diagnosis, Intervention 1 group)  3. Just that for me, I kind of zoned in on the emotional support around how you feel and how you react to the diagnosis, and thought I would get you know, more information and more of an insight. (Female, aged 31-40, undergoing diagnosis, Intervention 1 group) |
| Peer Support | Content of sessions | 1. I was quite angry all the time and I didn’t know why I was angry all the time. I didn’t understand what that was until again I spoke to the support worker there. I never really talked to anybody, and this has kind of made me have to talk to somebody I guess, otherwise I’d have been a mess. So that’s the biggest thing that I've taken from it, is it’s OK to talk about it. (Female, aged 31-40, 2 months since diagnosis, Intervention 2 group)  2. It’s the feeling, the outlook. It’s just – I’m young still, I’m 23 if I dare say that. And speaking to someone who’s a bit older, who’s – I think she [Peer Support Worker) was around 50-odd, and still, there’s nothing stopping her. MS isn’t stopping her – yeah. Better outlook on life. (Female, aged 21-30, 6 days since diagnosis, Intervention 2 group)  3. So she’s got experience in that way as well. So, she was saying if you need any advice in terms of like TIT and she had a lot of information on that, and all these things, so she said if you ever want any help with that, just let me know, and I’ve got all these numbers I can give you. It wasn’t just – obviously with her daughter with MS – but she also worked with the MS society, she just had all that experience there. (Female, aged 31-40, 1 week since diagnosis, Intervention 1 group)  4. They [patient-participant] found it [local MS Society support group] quite useful because, you know, if they didn’t they wouldn't continue […] like my first person, I think if he didn’t find that [support group] useful or if he didn’t like me or didn’t think it was worthwhile he’d just be like ‘no it’s OK’ and that would be it and maybe he wouldn't then come to our get togethers, etc., but because he stayed engaged, I believe that obviously that’s been successful. (Peer Support Worker) |

**Supplementary Material 5.** Fidelity of delivery of multiple sclerosis nurse specialist support.

| **Session recordings** | **Nurse check-in** | **Needs review** | **Outcomes identified** | **Support - information** | **Support - emotional** | **Refer workbook** | **Explain next steps** | **Discussions congruent** | ***Advice inconsistent*** | **Nurse flexibility** | **Session record form completed** | **Consistency form recording** | **Total** | **%** |
| --- | --- | --- | --- | --- | --- | --- | --- | --- | --- | --- | --- | --- | --- | --- |
| Recording 1 | 2 | 2 | 1 | 2 | 1 | 2 | 1 | 2 | 1 | 2 | 2 | 1 | **19** | 54 |
| Recording 2 | 1 | 1 | 1 | 1 | 0 | 0 | 0 | 1 | 1 | 1 | 1 | 1 | **9** | 26 |
| Recording 3 | 2 | 2 | 2 | 2 | 2 | 2 | 2 | 3 | 2 | 2 | 2 | 1 | **24** | 69 |
| Recording 4 | 2 | 2 | 2 | 2 | 2 | 2 | 1 | 3 | 2 | 2 | 2 | 2 | **24** | 69 |
| Recording 5 | 2 | 2 | 1 | 2 | 2 | 2 | 1 | 1 | 2 | 1 | 2 | 1 | **19** | 54 |
| Recording 6 | 2 | 2 | 2 | 2 | 2 | 2 | 2 | 3 | 2 | 2 | 2 | 1 | **24** | 69 |
| Recording 7 | 2 | 2 | 1 | 2 | 2 | 2 | 2 | 2 | 1 | 2 | 2 | 2 | **22** | 63 |
| Recording 8 | 0 | 2 | 1 | 2 | 2 | 2 | 1 | 2 | 2 | 2 | 2 | 2 | **20** | 57 |
| Recording 9 | 2 | 2 | 1 | 2 | N/A | 2 | 2 | 2 | 2 | 2 | 2 | 2 | **21** | 60 |
| Recording 10 | 2 | 2 | 1 | 2 | 2 | 1 | 1 | 2 | 2 | 1 | 0 | N/A | **16** | 46 |
| **Total** | **17** | **19** | **13** | **19** | **15** | **17** | **13** | **21** | **17** | **17** | **17** | **13** |  |  |

Key for scoring: 0 No; 1 Yes - Somewhat; 2 Yes - Mostly 3: Yes - Fully. ‘*Advice inconsistent*’ category was reverse scored: 0 Yes - Fully; 1 Yes - Somewhat; 2 No.

N/A: Not available.

**Supplementary Material 6.** Individual changes: Reliable and clinically significant changes - baseline to 3 months

| **Measure** | **Reference values** | **Usual care**  **n (%)** | | | **Intervention 1**  **Nurse-specialist support**  **n (%)** | | | **Intervention 2**  **Nurse-specialist and Peer support**  **n (%)** | | |
| --- | --- | --- | --- | --- | --- | --- | --- | --- | --- | --- |
|  |  | **No change** | **Improved** | **Deteriorated** | **No change** | **Improved** | **Deteriorated** | **No change** | **Improved** | **Deteriorated** |
| HADS anxiety | Mean reliability (Cronbach’s alpha) score of 0.83^12^; MS population^23^, cut-offs^24^ | 11 (85) | 2 (2*) (15) | 0 | 13 (100) | 0 | 0 | 6 (60) | 2 (1*) (20) | 2 (20) |
| HADS depression | Mean reliability (Cronbach’s alpha) 0.82^12^, MS population^13^, cut-offs^14^ | 11 (85) | 1 (1*) (8) | 1 (8) | 8 (67) | 1 (1*) (8) | 3 (25) | 11 (92) | 0 | 1 (8) |
| Perceived stress scale (PSS-4) | Mean reliability (Cronbach’s alpha) 0.84^15^, normative data^16^ | 10 (77) | 3 (2*) (23) | 0 | 9 (64) | 3 (3*) (21) | 2 (14) | 7 (58) | 3 (3*) (25) | 2 (17) |
| MS Self-efficacy scale (MSSE)^a^ |  | 1^b^ (10) | 7 (70) | 2 (20) | 0 | 5 (42) | 7 (58) | 0 | 5 (42) | 7 (58) |
| MSIS-Psy | Mean reliability (Cronbach’s alpha) 0.80^17^ | 11 (85) | 2 (15) | 0 | 11 (92) | 1 (8) | 0 | 10 (83) | 1 (8) | 1 (8) |

Reliable Change at p<0.05; *Clinically significant change; ^a^Reporting trends – an increase (positive) or decrease in individual scores; ^b^No change in scores. Hospital Anxiety and Depression Scale (HADS); Multiple Sclerosis (MS); Multiple Sclerosis Impact Scale-29 (MSIS-29)–psychological subscale (MSIS-psych); Multiple Sclerosis Self-Efficacy Scale (MSSE); Perceived Stress Scale 4-item (PSS-4).

**Supplementary Material 7.** Individual changes: Reliable and clinically significant changes – baseline to 6 months

| **Measure** | **Usual care**  **n (%)** | | | **Intervention 1**  **Nurse-specialist support**  **n (%)** | | | **Intervention 2**  **Nurse-specialist and Peer support**  **n (%)** | | |
| --- | --- | --- | --- | --- | --- | --- | --- | --- | --- |
|  | **No change** | **Improved** | **Deteriorated** | **No change** | **Improved** | **Deteriorated** | **No change** | **Improved** | **Deteriorated** |
| HADS anxiety | 10 (77) | 2 (2*) (15) | 1 (8) | 11 (84) | 1 (1*) (8) | 1 (8) | 7 (70) | 2 (1*) (20) | 1 (10) |
| HADS depression | 9 (70) | 2 (2*) (15) | 2 (15) | 10 (77) | 0 | 3 (23) | 10 (100) | 0 | 0 |
| Perceived stress scale (PSS-4) | 11 (84) | 1 (1*) (8) | 1 (8) | 7 (54) | 3 (3*) (23) | 3 (23) | 6 (60) | 2 (2*) (20) | 2 (20) |
| MS Self-efficacy scale (MSSE) ^a^ | 0 | 6 (60) | 4 (40) | 2^b^ (18) | 2 (18) | 7 (64) | 1^b^ (10) | 4 (40) | 5 (50) |
| MSIS-Psy | 11 (85) | 2 (15) | 0 | 11 (92) | 0 | 1 (8) | 8 (80) | 1 (10) | 1 (10) |

Reliable Change at p<0.05; *Clinically significant change; ^a^Reporting trends – an increase (positive) or decrease in individual scores; ^b^No change in scores, Hospital Anxiety and Depression Scale (HADS); Multiple Sclerosis: MS; Multiple Sclerosis Impact Scale-29 (MSIS-29)–psychological subscale (MSIS-psych); Multiple Sclerosis Self-Efficacy Scale (MSSE); Perceived Stress Scale 4-item (PSS-4).

**References (supplementary materials)**

1. Cohen J. *Statistical power analysis for the behavioral sciences*. 2nd Edition ed. New York: Routledge, 2013.
2. Avery KNL, Williamson PR, Gamble C, et al. Informing efficient randomised controlled trials: exploration of challenges in developing progression criteria for internal pilot studies. *BMJ Open*. 2017; 7: e013537. doi: <https://doi.org/10.1136/bmjopen-2016-013537>
3. Hoffmann TC, Glasziou PP, Boutron I, et al. Better reporting of interventions: template for intervention description and replication (TIDieR) checklist and guide. *BMJ*. 2014; 348. doi: <https://doi.org/10.1136/bmj.g1687>
4. Topcu G, Griffiths H, Bale C, et al. *Providing Emotional Support around the point of Multiple Sclerosis diagnosis (PrEliMS): Phase 1 report*. Nottingham: University of Nottingham, 2017.
5. Dennison L, Yardley L, Devereux A and Moss-Morris R. Experiences of adjusting to early stage Multiple Sclerosis. *J Health Psych*. 2011; 16: 478-88. doi: [10.1177/1359105310384299](https://doi.org/10.1177/1359105310384299)
6. Soundy A, Roskell C, Adams R, Elder T and Dawes H. Understanding health care professional-patient interactions in multiple sclerosis: A systematic review and thematic synthesis. *Open J Ther Rehabil.* 2016; 4: 187-217. doi: [10.4236/ojtr.2016.44018](http://dx.doi.org/10.4236/ojtr.2016.44018)
7. Wilkinson HR and das Nair R. The psychological impact of the unpredictability of multiple sclerosis: a qualitative literature meta-synthesis. *Br J Neurosci Nursing*. 2013; 9: 172-8. doi: [10.12968/bjnn.2013.9.4.172](https://doi.org/10.12968/bjnn.2013.9.4.172)
8. Mental Health Foundation. *Peer Support in Long Term Conditions: The Basics*. Edinburgh: Mental Health Foundation, 2013.
9. VISN 1 New England MIRECC Peer Education Centre and VISN 4 MIRECC Peer Resource Centre. *Peer Specialist Toolkit: Implementing Peer Support Services in VHA*. Washington: U.S. Department of Veterans Affairs, 2013.
10. Hayes SC, Strosahl K and Wilson KG. *Acceptance and commitment therapy: An experiential approach to behavior change*. New York: Guildford Press, 1999.
11. Carroll C, Patterson M, Wood S, Booth A, Rick J and Balain S. A conceptual framework for implementation fidelity. *Impl Sci*. 2007; 2: 40. doi: [10.1186/1748-5908-2-40](https://doi.org/10.1186/1748-5908-2-40)
12. Bjelland I, Dahl AA, Haug TT and Neckelmann D. The validity of the Hospital Anxiety and Depression Scale. An updated literature review. *J Psychosom Res*. 2002; 52: 69-77. DOI: [10.1016/S0022-3999(01)00296-3](https://doi.org/10.1016/S0022-3999(01)00296-3)

13. Jones KH, Ford DV, Jones PA, et al. A large-scale study of anxiety and depression in people with Multiple Sclerosis: a survey via the web portal of the UK MS Register. *PloS one*. 2012; 7: e41910. DOI: [10.1371/journal.pone.0041910](https://doi.org/10.1371/journal.pone.0041910)

14. Watson TM, Ford E, Worthington E and Lincoln NB. Validation of Mood Measures for People with Multiple Sclerosis. *Int J MS Care*. 2014; 16: 105-9. DOI: [10.7224/1537-2073.2013-013](https://doi.org/10.7224%2F1537-2073.2013-013)

15. Wu SM and Amtmann D. Psychometric Evaluation of the Perceived Stress Scale in Multiple Sclerosis. *Int Sch Res Notices*. 2013; 2013: 608356. DOI: [10.1155/2013/608356](https://doi.org/10.1155/2013/608356)

16. Warttig SL, Forshaw MJ, South J and White AK. New, normative, English-sample data for the Short Form Perceived Stress Scale (PSS-4). *J Health Psychol*. 2013; 18: 1617-28. DOI: [10.1177/1359105313508346](http://dx.doi.org/10.1177/1359105313508346)

17. McGuigan C and Hutchinson M. The multiple sclerosis impact scale (MSIS-29) is a reliable and sensitive measure. *J Neurol Neurosurg Psychiatry*. 2004; 75: 266.
